# Longevity Extension by Phytochemicals

**DOI:** 10.3390/molecules20046544

**Published:** 2015-04-13

**Authors:** Anna Leonov, Anthony Arlia-Ciommo, Amanda Piano, Veronika Svistkova, Vicky Lutchman, Younes Medkour, Vladimir I. Titorenko

**Affiliations:** Department of Biology, Concordia University, 7141 Sherbrooke Street, West, SP Building, Room 501-13, Montreal, QC H4B 1R6, Canada; E-Mails: anna_leonova@yahoo.com (A.L.); anthony.arlia@outlook.com (A.A.-C.); amandapiano91@hotmail.com (A.P.); klubnika_veronika@hotmail.com (V.S.); lutchman.david@hotmail.com (V.L.); writetoyounes@gmail.com (Y.M.)

**Keywords:** phytochemicals, interspecies chemical signals, aging, longevity regulation mechanisms, evolution, ecosystems, hormesis, signaling pathways, stress response, metabolism

## Abstract

Phytochemicals are structurally diverse secondary metabolites synthesized by plants and also by non-pathogenic endophytic microorganisms living within plants. Phytochemicals help plants to survive environmental stresses, protect plants from microbial infections and environmental pollutants, provide them with a defense from herbivorous organisms and attract natural predators of such organisms, as well as lure pollinators and other symbiotes of these plants. In addition, many phytochemicals can extend longevity in heterotrophic organisms across phyla via evolutionarily conserved mechanisms. In this review, we discuss such mechanisms. We outline how structurally diverse phytochemicals modulate a complex network of signaling pathways that orchestrate a distinct set of longevity-defining cellular processes. This review also reflects on how the release of phytochemicals by plants into a natural ecosystem may create selective forces that drive the evolution of longevity regulation mechanisms in heterotrophic organisms inhabiting this ecosystem. We outline the most important unanswered questions and directions for future research in this vibrant and rapidly evolving field.

## 1. Introduction

Plants use a diverse set of secondary biochemical pathways not to fulfill their primary metabolic needs in energy and biosynthetic products, but to generate a number of secondary metabolites called phytochemicals [1,2,3,4,5]. Some phytochemicals are produced not by plants, but by non-pathogenic endophytic bacteria and fungi that live within the plants [6,7,8,9,10,11]. 

Phytochemicals are structurally diverse chemical compounds; based on chemical nature, they can be divided into the following major classes: (1) phenolic compounds, including flavonoids, phenolic acids, hydroxycinnamic acids, lignans, tyrosol esters, stilbenoids and alkylresorcinols; (2) terpenes, including carotenoids, monoterpenes, saponins, some modified lipid species and triterpenoids; (3) betalains, including betacyanins and betaxanthins; (4) polysulfides; (5) organosulfur compounds; (6) indole compounds; (7) some protease inhibitors; (8) oxalic and anacardic organic acids; (9) modified purines; (10) quinones; and (11) polyamines [5,12,13,14,15,16,17,18]. It is believed that plants have evolved secondary biochemical pathways for synthesizing chemically diverse phytochemicals as ecosystemic adaptations; such evolutionary adaptations are thought to increase the chances of these immobile autotrophic organisms to survive and reproduce within their natural ecological niches [1,2,3,5,13,19,20,21]. Indeed, phytochemicals are used by plants as interspecies chemical signals that can: (1) help plants to survive various environmental stresses, including UV light, heat and cold stresses, osmotic stress and high salinity, extreme pH, water deficit and dehydration, and nutrient deprivation; (2) protect plants from viral, bacterial, yeast and fungal infections; (3) defend plants from invading insects, herbivorous animals and competitor plant species; (4) provide plants with a protection from environmental pollutants; (5) attract pollinators and other symbiotes; and (6) attract the natural predators of herbivorous insects and animals [1,2,5,13,19,20,21,22,23,24,25,26,27,28,29,30,31,32,33,34,35]. Moreover, phytochemicals that are produced by non-pathogenic endophytic microorganisms living within the plants can promote the survival of the host plants by protecting them from being eaten by herbivorous insects and animals as well as by defending them from many environmental stresses and infections by pathogenic microorganisms [6,7,8,9,10,11,36,37,38,39,40,41]. 

A body of evidence supports the notion that, in addition to being beneficial to survival and reproduction of the plants producing them, phytochemicals can extend longevity and/or improve health in various heterotrophic organisms [5,13,14,15,16,17,18,21,22,27,39,42,43,44,45,46,47,48,49,50,51,52,53,54,55,56,57,58,59,60]. In this review, we discuss recent progress in understanding mechanisms underlying such longevity-extending and health-improving effects of phytochemicals on heterotrophic organisms across phyla. We also propose a hypothesis in which phytochemicals that have been released by plants into an ecosystem create xenohormetic, hormetic and cytostatic selective forces that may drive the evolution of longevity regulation mechanisms in heterotrophic organisms inhabiting this ecosystem.

## 2. Phytochemicals Extend Lifespan in Evolutionarily Distant Heterotrophic Organisms by Targeting an Evolutionarily Conserved Set of Longevity-Defining Cellular Processes

Table 1 recapitulates numerous findings on how different phytochemicals prolong longevity in various heterotrophic organisms by modulating certain cellular processes [61,62,63,64,65,66,67,68,69,70,71,72,73,74,75,76,77,78,79,80,81,82,83,84,85,86,87,88,89,90,91,92,93,94,95,96,97,98,99,100,101,102,103,104,105,106,107,108,109,110,111,112,113,114,115]. The mechanisms by which these phytochemicals extend lifespan in organisms across phyla have begun to emerge. In this section, we discuss such mechanisms. 

### 2.1. Longevity-Extending Phytochemicals and Heterotrophic Organisms Whose Lifespans They Prolong

Longevity-extending phytochemicals differ in chemical nature; they belong to various classes, including phenolic compounds [61,66,67,70,72,74,75,76,77,79,81,82,83,84,86,89,90,91,92,93,94,95,96,97,98,99,100,101,102,103,106,107,108,109,114,115], terpenes [73,78,85,87], polysulfides [80], organosulfur compounds [62,63,64,65], indole compounds [88,104,105], modified purines [68,69,70,71], quinones [17] and polyamines [111] (Table 1). These phytochemicals extend lifespan in such evolutionarily distant heterotrophic organisms and cultured cells as the budding yeast *Saccharomyces cerevisiae* [17,66,68,97,98,111], the fission yeast *Schizosaccharomyces pombe* [69], the nematode *Caenorhabditis elegans* (including a transgenic model of Alzheimer’s disease) [67,70,71,72,74,80,81,82,83,84,85,86,87,89,90,99,100,101,103,104,105,106,111,114,115], the fruit fly *Drosophila* *melanogaster* (including different transgenic models of Alzheimer’s disease) [61,75,76,77,94,106,111], the honey bee *Apis mellifera* [108], mosquitoes [95], the naturally short-lived fish *Nothobranchius Furzeri* [107], laboratory mice (including mice on a high-calorie diet and transgenic mice models of several age-related diseases) [62,63,64,65,73,78,91,92,109], laboratory rats [93], different lines of cultured human fibroblasts [79,88,96,102], and human peripheral blood mononuclear cells [111] (Table 1). It needs to be emphasized that some studies revealed that several of the longevity-extending phytochemicals mentioned in Table 1 are unable to prolong lifespan in certain heterotrophic organisms; for example, such phenolic compounds as resveratrol and curcumin did not alter the lifespan in genetically heterogeneous mice [116]. 

### 2.2. Proteins and Signaling Pathways Required for Longevity Extension by Phytochemicals

Cellular proteins and signaling pathways that are indispensable for lifespan-prolonging abilities of many longevity-extending phytochemicals have been identified (Table 1). They include the following proteins and pathways: (1) DAF-2, the only known receptor of the insulin/insulin-like growth factor 1 (IGF-1) signaling (IIS) pathway; this pathway defines longevity of the nematode *C. elegans* by regulating metabolism, protein homeostasis, resistance to many stresses, development and reproduction [70,71,89,101,115]; (2) the phosphatidylinositol 3-kinase AGE-1, an essential protein component of the IIS pathway in the nematode *C. elegans* [74,99,100,101]; (3) AKT-2, a serine/threonine protein kinase involved in the IIS pathway in the nematode *C. elegans* [72]; (4) SKN-1/Nrf, one of the transcription factors playing an essential role in the IIS pathway in the nematode *C. elegans* [74,80]; (5) the heat-shock factor 1 (HSF-1), a transcriptional factor involved in the IIS pathway in the nematode *C. elegans* [89,115]; (6) the transcription factor DAF-16/FOXO and its nuclear import in the nematode *C. elegans*—this protein is a key component of the IIS pathway [67,70,71,81,86,89,90,99,100,101,114,115]; (7) the OSR-1/UNC-43 (CaMKII)/SEK-1 (p38 MAPK) signaling pathway, which in the nematode *C. elegans* defines resistance to osmotic stress, arsenic, and pathogen infection [67,74,101]; (8) the nicotinic acetylcholine receptor EAT-2, which is essential for longevity regulation in the nematode *C. elegans* because it defines the rate of pharyngeal pumping in this organism [70,80,81,114]; (9) NHR-8, a non-canonical nuclear hormone receptor which is essential for longevity regulation—likely because it defines resistance to xenobiotic stress and plays essential roles in the metabolism of cholesterol, bile acids, and neutral lipids in the nematode *C. elegans* [72]; (10) the mitogen-activated protein kinase (MAPK) kinase MEK-1, which in the nematode *C. elegans* is involved in protein synthesis and stress-induced apoptosis and defines resistance to pathogen infection and heavy metals [74]; (11) the MEV-1 subunit of succinate-coenzyme Q oxidoreductase, a component of the mitochondrial electron transport chain that defines longevity of the nematode *C. elegans* [70,72,81,114]; (12) the histone acetyl transferase CBP-1, a transcriptional activator which is involved in mRNA processing and neurogenesis in the nematode *C. elegans* [70,71]; (13) TPH-1, a tryptophan hydroxlase enzyme involved in serotonin synthesis in the nematode *C. elegans* [104]; (14) the sirtuins Sir1 in the yeast *S. cerevisiae* [66], SIR-2.1 in the nematode *C. elegans* [67,74,106], Sir2 in the fruit fly *D. melanogaster* [106], and SIRT1 in mice on a high-calorie diet [42,54,60,109]—all of which define longevity by modulating numerous cellular processes; (15) the target of rapamycin complex 1 (TORC1), which in the yeasts *S. cerevisiae* and *Sch. pombe* controls cell metabolism, protein synthesis, resistance to many stresses, and autophagy [17,68,69]; (16) the nutrient-sensing protein kinases Sch9 and Gcn2, which define longevity by modulating cell cycle progression, transcription, protein synthesis, responses to various stresses, amino acid synthesis and sphingolipid synthesis in the yeast *S. cerevisiae* [17]; (17) cytosolic and mitochondrial superoxide dismutases Sod1 and Sod2 (respectively), both playing essential roles in longevity regulation by detoxifying the superoxide radical, modulating cellular respiration, and controlling cell response to various stresses in the yeast *S. cerevisiae* [17,97]; and (18) the non-selective autophagy pathway for degradation of various cellular organelles and macromolecules in the yeast *S. cerevisiae*, nematode *C. elegans*, and fruit fly *D. melanogaster* [111,112,113].

### 2.3. Processes Targeted by Longevity-Extending Phytochemicals in Evolutionarily Distant Organisms

Longevity-extending phytochemicals have been shown to elicit changes in various cellular and organismal processes in organisms across phyla. These processes and organisms are outlined below and detailed in Table 1.

**Table 1:** Phytochemicals that extend lifespan in various heterotrophic organisms and longevity-defining cellular processes that they modulate. Abbreviations: CaMK, Ca^2+^/calmodulin-dependent protein kinase; FOXO, forkhead box protein O; HDTIC, 4-hydroxy-5-hydroxymethyl-[1,3]dioxolan-2,6'-spirane-5',6',7',8'-tetrahydro-indolizine-3'-carbaldehyde; Q3'G, quercetin 3'-O-β-d-glucopyranoside; Q3M, 3-O-β-d-glucopyranoside-(4→1)-β-d-glucopyranoside; MAPK, mitogen-activated protein kinase; NT, not tested; rDNA, ribosomal DNA; ROS, reactive oxygen species.

Phytochemical	Plant	Chemical Nature	Organism Exhibiting Lifespan Extension	Cellular Proteins and Signaling Pathways Required	Changes Caused
Acteoside	*Phlomis anisodonta*, *Phlomis bruguieri*, *Verbascum phlomoides*, *Verbascum mallophorum*, *Buddleja globose*, *Buddleja cordata*	Caffeoyl phenylethanoid glycoside (a phenolic compound)	The fruit fly *Drosophila melanogaster* [61]	NT	NT
Allicin	*Allium sativum* (garlic)	Organosulfur compound	• Senescence-accelerated mice [62,63,64,65]	NT	• Improved memory retention and acquisition [62,63,64,65]
Butein	*Toxicodendron vernicifluum*	Chalconoid (a phenolic compound)	• The yeast *Saccharomyces cerevisiae* [66]	• The sirtuin Sir1 [66]	NT
Caffeic acid, rosmarinic acid	*Eucalyptus globulus*, *Salvinia molesta*	Hydroxycinnamic acids (phenolic compounds)	• The nematode *Caenorhabditis elegans* [67]	• The OSR-1/UNC-43 (CaMKII)/SEK-1 (p38 MAPK) signaling pathway [67] • The sirtuin SIR-2.1 [67] • Caffeic acid only: the DAF-16/FOXO transcription factor [67]	• Lowered susceptibility to thermal stress [67] • Decreased oxidative damage to macromolecules [67] • Reduced body size, altered lipid metabolism, delayed reproductive timing [67]
Caffeine	*Coffea* plants	Methylxanthine (a purine)	• The yeasts *Saccharomyces cerevisiae* [68] and *Schizosaccharomyces pombe* [69] • The nematode *C. elegans* [70,71]	• In *S. cerevisiae* and *Sch. pombe*: the target of rapamycin complex 1 (TORC1) [68,69] • In *C. elegans*: the insulin-like receptor DAF-2, transcription factor DAF-16/FOXO and transcriptional activator CBP-1 [70,71]	• In *S. cerevisiae*: enhanced transcription of genes encoding heat-shock proteins and molecular chaperones [68] • In *Sch. pombe*: decelerated growth, G2 cell-cycle arrest, altered transcription of many nuclear genes, attenuated protein synthesis and inhibited phosphorylation of ribosomal S6 proteins [69] • In *C. elegans*: delayed onset of paralysis and reduced protein aggregation in nematode models of the Alzheimer’s and Huntington’s diseases [70,71]
Catechin	Vascular plants	Flavan-3-ol (a phenolic compound)	• The nematode *C. elegans* [72]	• The AKT-2 serine/threonine protein kinase, MEV-1 subunit of succinate-coenzyme Q oxidoreductase in the mitochondrial electron transport chain, and nuclear hormone receptor NHR-8 [72]	• Reduced body length and susceptibility to thermal stress [72] • Elevated pumping rate [72]
Celastrol	*Tripterygium wilfordii*, *Celastrus regelii*	Triterpenoid (a terpen)	• Transgenic mouse model of amyotrophic lateral sclerosis (ALS) [73]	NT	• Decelerated weight loss, improved motor performance, increased number of neurons and delayed onset of ALS [73]
Curcumin, tetrahydrocurcumin	*Curcuma longa*	Diarylheptanoids (phenolic compounds)	• The nematode *C. elegans* [74] • The fruit fly *D. melanogaster*, including 5 different models of Alzheimer’s disease [75,76,77]	• In *C. elegans*: the OSR-1/UNC-43 (CaMKII)/SEK-1 (p38 MAPK) signaling pathway [74] • In *C. elegans*: the sirtuin SIR-2.1 [74] • In *C. elegans*: the phosphatidylinositol 3-kinase AGE-1, transcription factor SKN-1/Nrf and MAPK kinase MEK-1 [74]	• In *C. elegans*: Reduced ROS levels, macromolecular oxidative damage, susceptibility to oxidative and thermal stresses, body length, and pumping rate [74] • In *D. melanogaster*: Decreased macromolecular oxidative damage, lowered susceptibility to oxidative stress, improved locomotor performance [75,76,77]
Crocin	*Crocus*, *Gardenia*	Carotenoid (a terpen)	• Dalton’s lymphoma ascites bearing mice [78]	NT	• Increased hemoglobin and lymphocytes [78] • Decreased white blood cell count and neutrophils [78]
Cryptotanshinone	*Salvia miltiorrhiza*	Tanshion (a quinone)	• The yeast *S. cerevisiae* [17]	• Mitochondrial superoxide dismutase Sod2, as well as the nutrient-sensing protein kinases Tor1, Sch9 and Gcn2 [17]	• Lowered ROS levels [17]
Cyanidin	*Vitis vinifera*, *Vitis labrusca*, *Vaccinium myrtillus*, *Vaccinium uliginosum*, *Vaccinium alaskaense*, *Vaccinium angustifolium*	Anthocyanidin (a phenolic compound)	• WI-38 human diploid fibroblasts [79]	NT	• Reduced oxidative damage to lipids and susceptibility to oxidative stress [79]
Diallyl trisulfide	*Allium sativum* (garlic)	A polysulfide (an organosulfide compound)	• The nematode *C. elegans* [80]	• The nicotinic acetylcholine receptor EAT-2 and transcription factor SKN-1/Nrf [80]	• Altered expression of many nuclear genes involved in metabolism and stress response [80]
Ellagic acid	*Quercus alba*, *Quercus robur*, *Myriophyllum spicatum*	Phenolic acid (a phenolic compound)	• The nematode *C. elegans* [81]	• The nicotinic acetylcholine receptor EAT-2 [81]	• Delayed beginning of egg deposition and reduced oxidative damage to water-soluble metabolites [81]
Epigallocatechin gallate	*Camellia sinensis*	Flavan-3-ol (a phenolic compound)	• The nematode *C. elegans* [82,83]	NT	• Lowered ROS levels, reduced susceptibility to oxidative stress, decreased oxidative damage to lipids, attenuated expression of nuclear genes encoding HSP-16, induced nuclear import of the transcription factor DAF-16/FOXO, reduced formation of Aβ deposits [82,83]
Epicatechin	Seed of *Theobroma cacao*, juice of *Prunus domestica*, seed of *Vicia faba*, oil from the fruit of *Euterpe oleracea*	Flavan-3-ol (a phenolic compound)	• The fruit fly *Drosophila melanogaster* [84] • Obese diabetic mice [84]	NT	• In obese diabetic mice: reduced degeneration of aortic vessels, lowered fat deposition, decreased hydropic degeneration in the liver, reduced markers of systematic inflammation, lowered serum LDL cholesterol, decreased level of circulating insulin-like growth factor 1, improved skeletal muscle stress output, increased concentration of hepatic glutathione, elevated superoxide dismutase activity, amplified AMP-activated protein kinase activity in the liver and skeletal muscle [84]
Ferulsinaic acid	*Ferula* plants	Sesquiterpene coumarin (a terpene)	• The nematode *C. elegans* [85]	NT	• Reduced susceptibility to oxidative and thermal stresses, decreased oxidative damage to lipids, lowered formation of advanced glycation end products [85]
Fisetin	*Acacia greggii*, *Acacia berlandieri*, *Butea frondosa*, *Gleditsia triacanthos*, *Quebracho colorado*, *Rhus cotinus*	Flavonol (a phenolic compound)	• The yeast *S. cerevisiae* [66] • The nematode *C. elegans* [86]	• In *S. cerevisiae*: The sirtuin Sir1 [66] • In *C. elegans*: Nuclear import of the transcription factor DAF-16/FOXO [86]	• In *S. cerevisiae*: NT [66] • In *C. elegans*: Lowered ROS levels, reduced susceptibility to oxidative stress, decreased oxidative damage to macromolecules, induced nuclear import of transcription factor DAF-16/FOXO [86]
Gallic acid	*Quercus alba*, *Quercus robur*, *Caesalpinia mimosoides*, *Boswellia dalzielii*, *Rhodiola rosea*, *Toona sinensis*	Phenolic acid (a phenolic compound)	• The nematode *C. elegans* [81]	• The nicotinic acetylcholine receptor EAT-2 [81]	• Increased body length, delayed beginning of egg deposition and reduced oxidative damage to water-soluble metabolites [81]
Glaucarubinone	*Simaroubaceae* plants	Triterpenoid (a terpen)	• The nematode *C. elegans* [87]	NT	• Increased rate of oxygen consumption and lowered levels of neutral lipids [87]
HDTIC-1, HDTIC-2	*Astragalus membranceus*	Indolizines (indole compounds)	• Human fetal lung diploid fibroblasts [88]	NT	• Improved growth and proliferation, accelerated entry from G0 or G1 phase to S phase, decreased activity of the senescence-associated-β-galactosidase, and reduced formation of advanced glycation end products [88]
Icariin, icariside II	*Epimedium plants*	Flavonol glycosides (phenolic compounds)	• The nematode *C. elegans* [89]	• The insulin-like receptor DAF-2, transcription factor DAF-16/FOXO and heat shock transcription factor HSF-1 [89]	• Reduced susceptibility to oxidative and thermal stresses, decelerated decline in age-related locomotion, delayed onset of paralysis caused by the proteotoxicity of polyQ and Aβ(1–42), enhanced transcription of the SOD-3 and HSP-12.3 genes [89]
Kaempferol	*Aloe vera*, *Coccinia grandis*, *Cuscuta chinensis*, *Euphorbia pekinensis*, *Glycine max*, *Hypericum perforatum*	Flavonol (a phenolic compound)	• The nematode *C. elegans* [86]	• Nuclear import of the transcription factor DAF-16/FOXO [86]	• Lowered ROS levels, reduced susceptibility to oxidative stress, decreased oxidative damage to macromolecules, induced nuclear import of transcription factor DAF-16/FOXO [86]
Myricetin	*Morella rubra*, *Myrica cerifera*, *Rosa damascene*, *Salvia hispanica*, *Hovenia dulcis*, *Ceratonia siliqua*	Flavonol (a phenolic compound)	• The nematode *C. elegans* [90]	• Nuclear import of the transcription factor DAF-16/FOXO [90]	• Lowered ROS levels, reduced oxidative damage to proteins, induced nuclear import of transcription factor DAF-16/FOXO, enhanced transcription of the SOD-3 gene [90]
Nordihydroguaiaretic acid	*Larrea tridentata*	Lignan (a phenolic compound)	• Transgenic mouse model of ALS [91] • Male mice [92] • Rats [93] • The fruit fly *D. melanogaster* [94] • Mosquitoes [95]	NT	• In transgenic mouse model of ALS: reduced motor dysfunction [91] • In *D. melanogaster*: lowered rate of oxygen consumption [94]
Oleuropein	*Olea europaea*	Phenylethanoid (a phenolic compound)	• Human embryonic fibroblasts [96]	NT	• Lowered ROS levels, reduced oxidative damage to proteins, increased rate of proteasomal degradation of oxidatively damaged proteins, decelerated age-related decline in proteasome activity [96]
Phloridzin	*Pyrus communis*, *Malus domestica*, *Prunus avium*, *Rosaceae* plants, *Dianthus caryophyllus*	Chalconoid (a phenolic compound)	• The yeast *S. cerevisiae* [97]	• Cytosolic and mitochondrial superoxide dismutases Sod1 and Sod2 (respectively) [97]	• Lowered ROS levels, decreased susceptibility to oxidative stress, activated transcription of the *SOD1*, *SOD2* and *SIR2* genes, increased superoxide dismutase activity [97]
Quercetin, Q3'G, Q3M, isorhamnetin, tamarixetin	*Capparis spinosa*, *Levisticum officinale*, *Rumex acetosa*, *Raphanus sativus*, *Ceratonia siliqua*, *Anethum graveolens*	Flavonols (phenolic compounds)	• The yeast *S. cerevisiae* [98] • The nematode *C. elegans* [67,99,100,101,103] • Human embryonic fibroblasts [102]	• In *C. elegans*: the insulin-like receptor DAF-2, phosphatidylinositol 3-kinase AGE-1 and nuclear import of the transcription factor DAF-16/FOXO [99,100,101], as well as the OSR-1/UNC-43 (CaMKII)/SEK-1 (p38 MAPK) signaling pathway [101]	• In *S. cerevisiae*: Lowered ROS levels, decreased glutathione oxidation, reduced protein carbonylation, lowered lipid peroxidation, decreased susceptibility to oxidative stress [98] • In *C. elegans*: Lowered ROS levels, reduced oxidative damage to macromolecules, enhanced anti-oxidative activities, decreased susceptibility to thermal and oxidative stresses, lowered level of neutral lipids, induced nuclear import of transcription factor DAF-16/FOXO [67,99,100,101] • In human fibroblasts: Lowered activity of the senescence-associated-β-galactosidase, decreased ROS levels, reduced susceptibility to oxidative stress, increased proteasome activity [102]
Reserpine	*Rauvolfia serpentina*	Indole alkaloid (an indole compound)	• The nematode *C. elegans* [104] • The nematode *C. elegans* model of Alzheimer’s disease [105]	• TPH-1, a tryptophan hydroxlase enzyme [104].	• Reduced susceptibility to thermal stress, decelerated decline in age-related locomotion and pharyngeal pumping, delayed postembryonic development [104,105] • The nematode *C. elegans* model of Alzheimer’s disease: delayed onset of paralysis caused by the proteotoxicity of Aβ [105]
Resveratrol	*Vitis* plants, *Vaccinium alaskaense*, *Vaccinium angustifolium*, *Rubus idaeus*, *Rubus occidentalis*, *Broussonetia papyrifera*	Stilbenoid (a phenolic compound)	• The yeast *S. cerevisiae* [66] • The nematode *C. elegans* [106] • The fruit fly *D. melanogaster* [106] • The short-lived fish *Nothobranchius Furzeri* [107] • The honey bee *Apis mellifera* [108] • Mice on a high-calorie diet [109]	• In *S. cerevisiae*: the sirtuin Sir1 [66] • The nematode *C. elegans*: the sirtuin SIR-2.1 [106] • The fruit fly *D. melanogaster*: the sirtuin Sir2 [106] • Mouse: SIRT1 and many other cellular targets, direct or indirect [42,54,60]	• In *S. cerevisiae*: reduced frequency of rDNA recombination [66] • In the nematode *C. elegans*: induced autophagy [110] • In *N. furzeri*: delayed age-related decay of locomotor activity and cognitive performances, reduced neurofibrillary degeneration in the brain [107] • In mouse: increased insulin sensitivity, increased activities of AMP-activated protein kinase (AMPK) and peroxisome proliferator-activated receptor-gamma coactivator 1α (PGC-1α), reduced levels of insulin-like growth factor-1 (IGF-I), increased number of mitochondria, altered transcription of many nuclear genes [109]
Spermidine, putrescine	Wheat (*Triticum* spp.) germ, *Ipomoea batatas*, *Pisum sativum, Glycine max*, *Glycine soja*	Polyamines	• The yeast *S. cerevisiae* [111] • The nematode *C. elegans* [111] • The fruit fly *D. melanogaster* [111] • Human peripheral blood mononuclear cells (PBMC) [111]	• In *S. cerevisiae*, *C. elegans* and *D. melanogaster*: autophagy [111,112]	• In *S. cerevisiae*, *D. melanogaster* and PBMC: lowered activities of histone acetyltransferases, increased histone H3 deacetylation, activated transcription of many autophagy-related genes, induced autophagy, delayed onset of age-related necrotic cell death, reduced age-related decline of locomotor activity [111] • In *D. melanogaster*: decelerated age-related decline of locomotor activity, increased level of triglycerides, altered relative levels of fatty acid species and phospholipid classes [112,113]
Tannic acid	*Caesalpinia spinosa*, *Rhus semialata*, *Quercus infectoria*, *Rhus coriaria*	Polyphenol (a phenolic compound)	• The nematode *C. elegans* [70,81,114]	• The mitogen-activated protein kinase kinase SEK-1, transcription factor DAF-16/FOXO, nicotinic acetylcholine receptor EAT-2 and MEV-1 subunit of succinate-coenzyme Q oxidoreductase in the mitochondrial electron transport chain [70,81,114]	• Reduced body length, decreased susceptibility to thermal and oxidative stresses, lowered levels of triglycerides, enhanced anti-oxidant capacity [70,81,114]
Tyrosol	Oil from the fruit of *Olea europaea*, oil from the kernels of *Argania spinosa*, leaves from *Camellia sinensis*	Phenylethanoid (a phenolic compound)	• The nematode *C. elegans* [115]	• The insulin-like receptor DAF-2, transcription factor DAF-16/FOXO and heat shock transcription factor HSF-1 [115]	• Decreased susceptibility to thermal and oxidative stresses, decelerated onset of age-related decline in pharyngeal pumping, activated transcription of nuclear genes encoding several heat-shock proteins [115]

#### 2.3.1. Yeasts

In the yeast *S. cerevisiae*, the changes elicited by longevity-extending phytochemicals include the following: (1) caffeine enhances transcription of genes encoding heat-shock proteins and molecular chaperones [68]; (2) cryptotanshinone reduces cellular levels of reactive oxygen species (ROS) [17]; (3) phloridzin decreases cellular levels of ROS, increases resistance to oxidative stress and superoxide dismutase activity, and activates transcription of the *SOD1* (cytosolic superoxide dismutase), *SOD2* (mitochondrial superoxide dismutase) and *SIR2* (sirtuin) genes [97]; (4) quercetin reduces cellular levels of ROS, the efficiencies of glutathione oxidation and lipid peroxidation, the extent of protein carbonylation, and cell susceptibility to oxidative stress [98]; (5) resveratrol decreases the frequency of rDNA recombination [66]; and (6) spermidine reduces activities of histone acetyltransferases, increases the extent of histone H3 deacetylation, activates transcription of many autophagy-related genes, induces autophagy and delays onset of age-related necrotic cell death [111] (Table 1). In the yeast *Sch. pombe*, caffeine decelerates growth, causes cell-cycle arrest in G2, alters transcription of many nuclear genes, attenuates protein synthesis and inhibits phosphorylation of ribosomal S6 proteins [69] (Table 1).

#### 2.3.2. The Nematode *C. elegans*

In the nematode *C. elegans*, longevity-extending phytochemicals cause the following changes: (1) caffeic and rosmarinic acids decrease susceptibility to thermal stress, reduce oxidative damage to macromolecules, lower body size, alter lipid metabolism and delay reproductive timing [67]; (2) caffeine delays the onset of paralysis and reduces protein aggregation in nematode models of Alzheimer’s and Huntington’s diseases [70,71]; (3) catechin lowers body length, reduces susceptibility to thermal stress, and elevates pumping rate [72]; (4) curcumin and tetrahydrocurcumin decrease cellular levels of ROS, the extent of oxidative damage to macromolecules, susceptibility to oxidative and thermal stresses, body length, and pumping rate [74]; (5) diallyl trisulfide alters expression of many nuclear genes involved in metabolism and stress response [80]; (6) ellagic acid delays the beginning of egg deposition and lowers the extent of oxidative damage to water-soluble metabolites [81]; (7) epigallocatechin gallate lowers cellular levels of ROS, reduces susceptibility to oxidative stress, decreases the extent of oxidative damage to lipids, attenuates expression of nuclear genes encoding HSP-16, enhances nuclear import of the transcription factor DAF-16/FOXO, and mitigates the formation of Aβ deposits [82,83]; (8) ferulsinaic acid reduces susceptibility to oxidative and thermal stresses, lowers the extent of oxidative damage to lipids, and slows down the formation of advanced glycation end products [85]; (9) fisetin decreases cellular levels of ROS, lowers susceptibility to oxidative stress, reduces the extent of oxidative damage to macromolecules and stimulates nuclear import of the transcription factor DAF-16/FOXO [86]; (10) gallic acid increases body length, delays the beginning of egg deposition, and reduces the extent of oxidative damage to water-soluble metabolites [81]; (11) glaucarubinone increases the rate of oxygen consumption and reduces cellular levels of neutral lipids [87]; (12) icariin and icariside II lower susceptibility to oxidative and thermal stresses, decelerate age-related decline in locomotion, delay the onset of paralysis elicited by the proteotoxicity of polyQ and Aβ(1–42), and stimulate transcription of the *SOD-3* and *HSP-12.3* genes [89]; (13) kaempferol lowers cellular levels of ROS, reduces susceptibility to oxidative stress, decreases the extent of oxidative damage to macromolecules, and accelerates nuclear import of the transcription factor DAF-16/FOXO [86]; (14) myricetin decreases cellular levels of ROS, lowers the extent of oxidative damage to proteins, stimulates nuclear import of the transcription factor DAF-16/FOXO and enhances transcription of the *SOD-3* gene [90]; (15) quercetin lowers cellular levels of ROS, decreases the extent of oxidative damage to macromolecules, elevates anti-oxidative activities, reduces susceptibility to thermal and oxidative stresses, reduces cellular levels of neutral lipids, and stimulates nuclear import of the transcription factor DAF-16/FOXO [67,99,100,101]; (16) reserpine decreases susceptibility to thermal stress, decelerates the age-related declines in locomotion and pharyngeal pumping, and delays postembryonic development [104,105]; in the nematode model of Alzheimer’s disease it also postpones the onset of paralysis caused by the proteotoxicity of Aβ [105]; (17) resveratrol and spermidine induce autophagy [110,111]; (18) tannic acid decreases body length, lowers susceptibility to thermal and oxidative stresses, reduces cellular levels of triglycerides, and enhances anti-oxidant capacity [70,81,114]; and (19) tyrosol lowers susceptibility to thermal and oxidative stresses, decelerates the onset of an age-related decline in pharyngeal pumping, and stimulates transcription of nuclear genes encoding several heat-shock proteins [115] (Table 1).

#### 2.3.3. The Fruit Fly *D. melanogaster*

In the fruit fly *D. melanogaster*, the alterations caused by longevity-extending phytochemicals include the following: (1) curcumin and tetrahydrocurcumin lower the extent of macromolecular oxidative damage, reduce susceptibility to oxidative stress, and improve locomotor performance [75,76,77]; (2) nordihydroguaiaretic acid decreases the rate of oxygen consumption [94]; and (3) spermidine lowers susceptibility to oxidative stress, induces autophagy, decelerates age-related decline of locomotor activity, increases cellular levels of triglycerides, and alters relative levels of fatty acid species and phospholipid classes [112,113] (Table 1).

#### 2.3.4. The Fish *Nothobranchius Furzeri*

In the naturally short-lived fish *N. furzeri*, resveratrol delays age-related decay of locomotor activity and cognitive performances [107]. This phenolic phytochemical is also known to reduce neurofibrillary degeneration in the brain of *N. furzeri* [107] (Table 1).

#### 2.3.5. Laboratory Mouse

In laboratory mice (including transgenic mice models of several age-related diseases and mice on a high-calorie diet), longevity-extending phytochemicals elicit the following changes: (1) allicin improves memory retention and acquisition in senescence-accelerated mice models [62,63,64,65]; (2) celastrol decelerates weight loss, improves motor performance, increases the number of neurons and delays the onset of amyotrophic lateral sclerosis (ALS) in a transgenic mouse model of ALS [73]; (3) crocin increases hemoglobin and lymphocytes, and decreases white blood cell count and neutrophils in Dalton’s lymphoma ascites-bearing mice [78]; (4) epicatechin reduces degeneration of aortic vessels and fat deposition, decreases hydropic degeneration in the liver and markers of systematic inflammation, lowers levels of serum LDL cholesterol and circulating insulin-like growth factor 1, improves skeletal muscle stress output, increases concentration of hepatic glutathione and total superoxide dismutase activity, and elevates AMP-activated protein kinase activity in diabetic mice [84]; (5) nordihydroguaiaretic acid reduces motor dysfunction in a transgenic mouse model of ALS [91]; and (6) resveratrol increases insulin sensitivity, stimulates activities of AMP-activated protein kinase (AMPK) and peroxisome proliferator-activated receptor-gamma coactivator 1α (PGC-1α), lowers levels of insulin-like growth factor-1 (IGF-I), elevates the number of mitochondria, and alters transcription of many nuclear genes in mice on a high-calorie diet [109] (Table 1).

#### 2.3.6. Cultured Human Cells

In cultured human cells, the alterations caused by longevity-extending phytochemicals include the following: (1) cyanidin lowers oxidative damage to lipids and decreases susceptibility to oxidative stress in WI-38 human diploid fibroblasts [79]; (2) two 4-hydroxy-5-hydroxymethyl-[1,3]dioxolan-2,6'-spirane-5',6',7',8'-tetrahydro-indolizine-3'-carbaldehydes (HDTIC), HDTIC-1, and HDTIC-2 improve growth and proliferation, accelerate entry from G0 or G1 phase to S phase of the cell cycle, lower activity of the senescence-associated-β-galactosidase, and decrease formation of advanced glycation end products in human fetal lung diploid fibroblasts [88]; (3) oleuropein lowers cellular levels of ROS, reduces oxidative damage to proteins, increases the rate of proteasomal degradation of oxidatively damaged proteins, and decelerates age-related decline in proteasome activity in human embryonic fibroblasts [96]; (4) quercetin lowers the activity of the senescence-associated-β-galactosidase, decreases cellular levels of ROS, reduces susceptibility to oxidative stress, and stimulates proteasome activity in human embryonic fibroblasts [102]; and (5) spermidine lowers the extent of histone H3 acetylation in human peripheral blood mononuclear cells (PBMC) and induces autophagy in human HeLa cells [111] (Table 1).

### 2.4. Mechanisms of Longevity Extension by Phytochemicals Are Evolutionarily Conserved

Findings described above in this section strongly support the notion that the mechanisms by which phytochemicals extend longevity of various heterotrophic organisms have been conserved in the course of evolution. Indeed, longevity-extending phytochemicals increase lifespan of such evolutionarily distant organisms as yeasts, worms, flies, bees, mosquitoes, fishes, laboratory mice, and laboratory rats [17,61,62,63,64,65,66,67,68,69,70,71,72,73,74,75,76,77,78,80,81,82,83,84,85,86,87,89,90,91,92,93,94,95,97,98,99,100,101,103,104,105,106,107,108,109,111,112,115]; these phytochemicals also prolong the replicative lifespans of different lines of cultured human cells [79,88,96,102,111] (Table 1). Furthermore, lifespan-prolonging abilities of these phytochemicals rely on cellular proteins integrated into several evolutionarily conserved signaling pathways known to regulate longevity in organisms across phyla [52,117,118,119,120,121,122,123,124,125,126]. These nutrient-, energy- and stress-sensing pathways include the following: (1) the IIS pathway [67,70,71,72,74,80,81,86,89,90,99,100,101,114,115]; (2) the TOR pathway [17,68,69]; (3) the sirtuin-governed protein deacetylation module of the longevity signaling network integrating the IIS and TOR pathways [45,54,60,66,67,74,106,109]; (4) the OSR-1/UNC-43 (CaMKII)/SEK-1 (p38 MAPK) stress-responsive signaling pathway [67,74,101]; and (5) the non-selective autophagy pathway for degradation of various cellular organelles and macromolecules [111,112,113] (Table 1). Moreover, these lifespan-prolonging phytochemicals postpone the onset of several longevity-defining cellular processes called “the cellular and molecular hallmarks of aging” [52,117,119,121,122,124,127,128,129,130,131,132,133]. Out of the nine commonly accepted cellular and molecular hallmarks of aging [124], lifespan-prolonging phytochemicals are known to delay the development of the following seven common traits of aging in evolutionarily distant heterotrophic organisms: (1) genomic instability [66]; (2) epigenetic alterations [111]; (3) loss of proteostasis [67,68,70,71,73,74,75,76,77,79,81,82,83,84,85,86,88,89,90,91,96,98,102,105,110,111,115]; (4) deregulated nutrient sensing [67,70,71,74,86,89,109]; (5) mitochondrial dysfunction [17,72,74,82,83,86,87,90,94,96,97,98,102,109]; (6) cellular senescence [62,63,64,65,69,88,102]; and (7) altered intercellular communication [62,63,64,65,73,78,107,112] (Table 1).

## 3. Phytochemicals: Interspecies Chemical Signals That May Contribute to the Evolution of Longevity Regulation Mechanisms within Natural Ecosystems

Findings summarized in the previous section provided the comprehensive evidence that many phytochemicals can extend lifespans of heterotrophic organisms across phyla via evolutionarily conserved mechanisms. These findings gave rise to a hypothesis on how such lifespan-extending capabilities of phytochemicals may contribute to the evolution of longevity regulation mechanisms in various organisms inhabiting a natural ecosystem. In this section, we discuss and extend this hypothesis.

### 3.1. The “Xenohormesis” Hypothesis 

The term “hormesis” has been introduced to define a special kind of response of cells and organisms to different doses of a stress agent. In this kind of stress response: (1) an exposure of a cell or an organism to low (“hormetic”) doses of a stress agent stimulates its growth, proliferation and/or survival; whereas (2) high doses of the same stress agent exhibit adverse effects on growth, proliferation and/or survival of this cell or organism [134,135,136,137]. Graphically, hormetic stress response is defined by a nonlinear and biphasic dose-response curve, which could be U-shaped, inverted U-shaped or J-shaped [44,57,138,139]. It is commonly accepted that an exposure of a cell or an organism to low doses of a hormetic stress agent elicits certain adaptive changes; by preconditioning the cell or the organism to a moderate stress, such changes can help to protect it against higher doses of the same (or related) stress agent [44,57,138,139,140].

The xenohormesis hypothesis posits that plants synthesize phytochemicals, in part, as a response to such hormetic environmental stresses as UV light, heat and cold stresses, osmotic stress and high salinity, water deficit and dehydration, nutrient deprivation, and infection [66,141,142]. Within the plant that synthesizes such phytochemicals, these secondary metabolites present at the concentrations that are not toxic but create a mild stress. Thus, within the host plant, such phytochemicals function as hormetic stress agents capable of inducing certain defense systems; these systems protect the plant against higher doses of the environmental stress that caused the synthesis of the phytochemicals and, possibly, against related environmental stresses [66,141,142]. According to the xenohormesis hypothesis, after the plant has released the phytochemicals into the ecosystem, these secondary metabolites (1) do not have any hormetic effect on the heterotrophic organisms inhabiting the ecosystem (and, thus, act as hormetic stress agents only within the host plant)—likely because the concentration of the phytochemicals outside the plant is below a hormetic threshold; (2) provide the heterotrophic organisms within the ecosystem with chemically encoded information on various changes in the environmental conditions taking place in the ecosystem; and (3) operate as interspecies chemical signals that can extend lifespans of various heterotrophic organisms within the ecosystem via evolutionarily conserved mechanisms, as described in the previous section [66,141,142]. The xenohormesis hypothesis postulates that the ability of heterotrophic organisms to remodel their metabolism and physiology in response to phytochemicals as messages on environmental changes within the ecosystem will increase their chances of survival, thus creating selective forces aimed at maintaining such ability [66,141,142]. Because many of these phytochemicals are also known to extend longevity in heterotrophic organisms across phyla by targeting evolutionarily conserved mechanisms, these selective forces may provide a mode of selection for the most efficient longevity regulation mechanisms [66,141,142]. Thus, phytochemicals may function as xenohormetic stress signals that drive the ecosystemic evolution of such mechanisms in heterotrophic organisms. 

### 3.2. Many Observations Contradict the Xenohormesis Hypothesis

One of the predictions of the xenohormesis hypothesis is that phytochemicals do not act as hormetic stress agents in heterotrophic organisms inhabiting the ecosystem [66,141,142]. However, recent findings imply that the dose-response effect of some phytochemicals on the longevity of heterotrophic organisms can be graphically described as an inverted U-shaped curve; these phytochemicals include caffeic acid, caffeine, ellagic acid, epigallocatechin gallate, quercetin, rosmarinic acid, and tannic acid [43,44,67,71,81,141]. Thus, at least caffeic acid, caffeine, ellagic acid, epigallocatechin gallate, quercetin, rosmarinic acid, and tannic acid can elicit a hormetic stress response in heterotrophic organisms; these phytochemicals are likely to operate as hormetic stress agents within both the host plants synthesizing them and heterotrophic organisms exposed to them. 

Furthermore, some phytochemicals exhibit moderate cytostatic effects in heterotrophic organisms across phyla. These phytochemicals include resveratrol and caffeine; they both act as cytostatic agents that retard cellular and organismal growth because of their abilities to attenuate the pro-aging TOR signaling pathway, a key driver of proliferative growth in heterotrophic organisms [68,126,143,144,145,146,147,148,149].

Moreover, some phytochemicals are not secondary metabolites that exist at low concentrations and are synthesized in secondary biochemical pathways, but primary metabolites that are relatively abundant and synthesized in primary biochemical pathways—one example of such phytochemicals is unsaturated fatty acids synthesized by plants in response to cold stress [13,150,151]. After being consumed by mammals, these unsaturated fatty acids are incorporated into cellular membranes in substantial quantities, thereby increasing membrane fluidity; this, in turn, elicits a longevity-extending process of heat shock response aimed at enhancing such age-delaying process as proteostasis maintenance [13,152,153].

### 3.3. An Extended Hypothesis on the Role of Phytochemicals in the Ecosystemic Evolution of Longevity Regulation Mechanisms

Because of the above contradictions to some projections of the xenohormesis hypothesis, we extend it by proposing a hypothesis in which phytochemicals that have been released by plants into a natural ecosystem may create xenohormetic, hormetic and cytostatic selective forces driving the evolution of longevity regulation mechanisms in heterotrophic organisms within this ecosystem. Our extended hypothesis posits that phytochemicals creating such selective forces after being released by plants into the ecosystem: (1) are either low-abundance chemical compounds formed by plants in secondary biochemical pathways or high-abundance products of primary biochemical pathways; (2) do not act as hormetic stress agents in heterotrophic organisms inhabiting the ecosystem or, alternatively, can trigger hormetic stress response in these organisms; (3) do not slow down growth of heterotrophic organisms within the ecosystem or, alternatively, cause a moderate delay of such growth by attenuating the pro-aging TOR signaling pathway; and (4) can increase lifespans of heterotrophic organisms inhabiting the ecosystem by modulating several evolutionarily conserved signaling pathways regulating longevity in these organisms; these signaling pathways and mechanisms of their modulation by phytochemicals have been described in the previous section. 

## 4. Conclusions and Future Perspectives 

Growing evidence supports the view that phytochemicals prolong lifespan in heterotrophic organisms across phyla by modulating a complex network of evolutionarily conserved signaling pathways; these pathways orchestrate a compendium of longevity-defining cellular processes that have been conserved in the course of evolution. The molecular and cellular mechanisms by which structurally diverse phytochemicals extend longevity of various heterotrophic organisms have emerged. Based on these findings, a hypothesis has been proposed on how the release of phytochemicals by plants into a natural ecosystem may create selective forces that guide the evolution of longevity regulation mechanisms in heterotrophic organisms inhabiting this ecosystem. The major challenge now is to empirically validate this hypothesis, perhaps by conducting experimental evolution of longevity regulation mechanisms in naturally short-lived heterotrophic organisms (such as the yeast *S. cerevisiae*, nematode *C. elegans*, fruit fly *D. melanogaster,* and fish *N. furzeri*) exposed to phytochemicals under laboratory conditions that mimic the natural stressful environment of cyclical starvation. It will be interesting to see if such long-term exposure of these organisms to phytochemicals can lead to selection of species that live longer than their predecessors. If such laboratory-evolved long-lived species can be selected, it will be intriguing to measure the relative fitness of each of them in a direct competition assay with their relatively short-lived ancestors. These experiments will provide an empirical validation test of the numerous evolutionary theories of aging trying to explain how the evolutionary force actively limits organismal lifespan at an age unique to each species [154,155,156,157,158,159,160,161,162,163].
